# Cognitive Impairment in People with Diabetes-Related Foot Ulceration

**DOI:** 10.3390/jcm10132808

**Published:** 2021-06-25

**Authors:** Ranita Siru, Melanie S. Burkhardt, Wendy A. Davis, Jonathan Hiew, Laurens Manning, Jens Carsten Ritter, Paul E. Norman, Ashley Makepeace, Peter Gerry Fegan, David G. Bruce, Timothy M. E. Davis, Emma J. Hamilton

**Affiliations:** 1 Department of Endocrinology and Diabetes, Fiona Stanley Hospital, Murdoch 6150, Australia; ranita.siru@gmail.com (R.S.); melanie.burkhardt@health.wa.gov.au (M.S.B.); ashley.makepeace@health.wa.gov.au (A.M.); gerry.fegan@health.wa.gov.au (P.G.F.); 2 Department of Clinical Psychology and Clinical Neuropsychology, Fiona Stanley and Fremantle Hospitals Group, Murdoch 6150, Australia; 3 Medical School, Fiona Stanley Hospital, University of Western Australia, Murdoch 6150, Australia; laurens.manning@uwa.edu.au (L.M.); paul.norman@uwa.edu.au (P.E.N.); 4 Medical School, Fremantle Hospital, University of Western Australia, Fremantle 6160, Australia; wendy.davis@uwa.edu.au (W.A.D.); david.bruce@uwa.edu.au (D.G.B.); tim.davis@uwa.edu.au (T.M.E.D.); 5 Department of Podiatry, Fiona Stanley Hospital, Murdoch 6150, Australia; jonathan.hiew@health.wa.gov.au; 6 Multidisciplinary Diabetes Foot Unit, Fiona Stanley and Fremantle Hospitals Group, Murdoch 6150, Australia; jens.ritter@health.wa.gov.au; 7 Department of Infectious Diseases and Microbiology, Fiona Stanley Hospital, Murdoch 6150, Australia; 8 Department of Vascular Surgery, Fiona Stanley Hospital, Murdoch 6150, Australia; 9 Faculty of Health Sciences, Curtin University, Bentley 6102, Australia

**Keywords:** diabetes-related foot ulcer, type 2 diabetes, cognition, self-care

## Abstract

Aims: To determine whether there is an excess of cognitive impairment in patients with type 2 diabetes and foot ulceration. Methods: 55 patients with type 2 diabetes and foot ulcers attending Multidisciplinary Diabetes Foot Ulcer clinics (MDFU cohort) were compared with 56 patients with type 2 diabetes attending Complex Diabetes clinics (CDC cohort) using commonly used screening tests for cognitive impairment (Mini-Mental State Examination (MMSE) and Montreal Cognitive Assessment (MOCA)), as well as foot self-care, mood and health literacy. MMSE was also compared between the MDFU cohort and a historical community-based cohort of patients with type 2 diabetes (FDS2 cohort). Results: Median MMSE scores were the same in all three groups (28/30). Median MOCA scores did not differ between the MDFU and CDC cohorts (25/30). There were no significant differences in the percentages of patients with MMSE ≤ 24 or MOCA ≤ 25 between MDFU and CDC cohorts (3.6% versus 10.7%, *p* = 0.27 and 56.4% versus 51.8%, *p* = 0.71, respectively), findings that did not change after adjustment for age, sex, education, diabetes duration, and random blood glucose. Conclusions: Using conventionally applied instruments, patients with type 2 diabetes and foot ulceration have similar cognition compared with patients without, from either hospital-based clinic or community settings.

## 1. Introduction

An individual with diabetes is estimated to have a 15–34% lifetime risk of developing a foot ulcer [1,2,3]. The potential consequences of a persistent diabetes-related foot ulcer (DFU) include lower extremity amputation (LEA) and increased mortality [4], and a heavy burden on the healthcare system [5,6]. Effective self-management strategies, including regular foot self-inspection, use of protective footwear that fits well, and maintenance of non-weight bearing status, are vital to positive outcomes [3,7]. Unfortunately, non-adherence to these measures is common [8,9,10] with patients reporting that they find management of DFUs disruptive and difficult [10,11,12].

International guidelines recommend proven preventive and self-management practices, optimal adherence to which should be achieved through patient education and participation in an integrated foot care program [13]. Effective education relies on the participant being capable of learning, understanding, remembering, and applying new information [6]. In relation to DFU, qualitative research has demonstrated a gap between patient knowledge of recommendations and the understanding of how they should be applied [10]. One possible explanation for this disparity may be a greater prevalence of cognitive impairment. Diabetes is an established risk factor for cognitive dysfunction and dementia [14,15]; these can occur relatively early in type 2 diabetes [16] and they share common risk factors with foot ulceration [17], such as hypertension [18], obesity, poor glycaemic control, diabetic retinopathy [19], and cerebrovascular disease [16]. Depression and anxiety are also common amongst patients with DFU, and may contribute to cognitive deficits [20,21]. Furthermore, it is possible that both cognitive dysfunction and DFU in people with diabetes share common mechanisms underlying vascular and neurological degeneration, including hyperglycaemia, altered insulin signalling, effects of advanced glycation, and chronic inflammation [22].

Only two studies have directly compared cognition in participants with diabetes with or without DFUs [6,17]. In the first, cognitive scores were significantly lower in those with a DFU but the specialized neurocognitive assessment tools used detected subtle cognitive deficits and there was an imbalance in risk factors between the groups [6]. In the second, Mini-Mental State Examination (MMSE) scores were lower in the patients with DFU compared to patients with diabetes without DFU and healthy controls, but there was a greater proportion of patients with hypertension, prior cardiovascular events, and dyslipidemia in the DFU group [17].

The presence of DFU-specific cognitive changes would be an important consideration in the development of tailored strategies to improve ulcer healing and avoid lower limb amputation in this vulnerable patient group. The primary aim of the present study was, therefore, to compare MMSE and Montreal Cognitive Assessment (MOCA) scores in individuals with type 2 diabetes attending a multidisciplinary diabetes foot ulcer clinic (MDFU) in those with type 2 diabetes but no foot ulceration attending a complex diabetes clinic (CDC). The secondary aim was to compare MMSE scores in the MDFU group to those in a matched group of people with type 2 diabetes participating in a longitudinal community-based observational study (Fremantle Diabetes Study (FDS2)).

## 2. Materials and Methods

The present study was an observational cross-sectional study conducted in the outpatient clinics of Fiona Stanley (FSH) and Fremantle Hospitals (FH), which are located in the southern suburbs of Perth, Western Australia. Participant recruitment and assessment commenced on 27 June 2018 and was completed on 3 July 2019. Participants with type 2 diabetes with an active, or recently healed (within three months), DFU were recruited from MDFU clinics providing intensive interdisciplinary care involving endocrinologists, vascular surgeons, infectious diseases physicians, and podiatrists. Participants with type 2 diabetes and no foot ulceration were recruited from the CDCs at each hospital. Patients aged 18–90 years with (i) type 2 diabetes mellitus, (ii) no known prior history of cognitive impairment, and (iii) fluency in English were eligible for recruitment. All participants provided written informed consent. The study was approved by the South Metropolitan Health Service Human Research Ethics Committee (RGS0000000457).

### 2.1. Clinical Methods

Each participant underwent a 90-min comprehensive assessment at a single visit. Details relating to diabetes, medical, and social history were obtained using a standardized questionnaire which included diabetes duration, treatment regimen, complications, comorbidities, smoking and alcohol consumption, marital status, educational level, and occupational history. Additionally, subjects underwent assessments of cognitive impairment, mood, health literacy, and foot self-care, as well as a physical examination and blood and urine tests. All questionnaires and physical examinations were performed by endocrinologists (R.S. or E.J.H.).

Assessment of cognitive impairment was performed using both the MMSE and MOCA. These were chosen for brevity, ease of use in a clinic setting, and familiarity to most clinicians. The MMSE, which comprises 30 questions covering four cognitive domains of orientation, attention, memory, and language, is a commonly used, well-validated screening test for clinically relevant cognitive impairment and dementia. A score ≤ 24 out of a maximum of 30 was taken to represent impaired cognition [23]. The MOCA is also composed of 30 questions and, in addition to the domains covered by the MMSE, assesses visuospatial ability, executive function, phonemic and syntactic fluency, and verbal abstraction. Participants scoring ≤ 25 were considered to have cognitive impairment [24]. The MOCA may be more sensitive than MMSE for detection of mild cognitive impairment in individuals with type 2 diabetes [21]. The internal consistency of the MMSE is considered adequate but may vary depending on the clinical context, with a high alpha level reported in a sample of mixed medical patients with more modest alpha described in community-based patient samples [25]. The internal consistency of the MOCA is good, with a Cronbach’s alpha of 0.83 reported by Nasreddine et al. [24].

Foot self-care was assessed using the Nottingham Assessment of Functional Foot Care (NAFF) [26]. The NAFF was developed as a surrogate outcome measure for trials of educational interventions in patients with diabetes-related foot disease. The original NAFF questionnaire comprised 29 items gauging the frequency of both beneficial and detrimental foot self-care behaviours, such as use of protective footwear and soaking the feet, respectively. Each item was scored from 0 to 3 and then summed to produce an overall score. The updated (2015) version of the NAFF questionnaire, comprising 26 items, was used in the present study. The NAFF has been shown to demonstrate good construct validity, test-retest reliability, and acceptable internal consistency [26].

As depression and anxiety symptoms may contribute to cognitive dysfunction [21], participants were screened for depression and anxiety using the four-item Patient Health Questionnaire for Depression and Anxiety (PHQ-4) [27]. This tool has been developed as a useful brief screening instrument with good internal reliability, construct validity, and factorial validity [27]. Since inadequate health literacy has also been identified as a major barrier to self-care in people with diabetes [28], this was assessed using the Brief Evaluation of Health Literacy. This questionnaire contains three items, each of which is capable of effectively detecting inadequate health literacy [29].

### 2.2. Fremantle Diabetes Study Phase 2 Comparison Cohort

In order to compare cognitive impairment in patients with type 2 diabetes and foot ulceration with community dwelling patients with type 2 diabetes, 55 participants matched for age, sex, and diabetes duration were selected from the Fremantle Diabetes Study Phase 2 (FDS2). The FDS2 is a longitudinal observational study of 1732 community-based participants recruited between 2008 and 2011 from an urban postcode-defined population of 157,000 in the state of Western Australia (WA) [30]. Of these, 1551 (89.5%) had type 2 diabetes and detailed clinical and biochemical assessment, MMSE (but not MOCA) and PHQ4 depression scores were available from the baseline visit. Details of recruitment, characterisation of diabetes, and non-recruited patients have been published [30]. The FDS2 protocol was approved by the Fremantle Hospital Human Research Ethics Committee and informed consent was obtained from each participant.

### 2.3. Laboratory Tests

Non-fasting blood and urine samples were collected for measurement of glucose level, glycated haemoglobin (HbA_1c_), urea, electrolytes, creatinine, lipid profile, and urine albumin:creatinine ratio (uACR) in a single accredited laboratory (Pathwest). Urine albumin and creatinine, and plasma glucose, creatinine, HDL-C, and triglycerides were assayed on the Architect C16000 automated analyzer (Abbott Laboratories, Abbott Park, IL, USA). LDL-C was calculated using the Friedewald formula. HbA_1c_ was measured in whole blood by immunoassay on the Roche Cobas Integra 800 (Roche Diagnostics, Mannheim, Germany). Total coefficient of variation was <6% for all analytes.

### 2.4. Statistical Analysis

The computer package IBM SPSS Statistics 25 (IBM Corporation, Armonk, NY, USA) was used for statistical analysis. Data are presented as proportions, mean ± SD, geometric mean (SD range), or, in the case of variables which did not conform to a normal or log-normal distribution, median and inter-quartile range (IQR). For independent samples, two-sample comparisons were by Fisher’s exact test for proportions, Student’s *t*-test for normally distributed variables, and Mann-Whitney *U*-test for non-parametric variables. Logistic regression modelling was undertaken with cognition as the outcome and MDFU versus CDC attendance the exposure of interest with (i) no adjustment, (ii) adjustment for age and sex, and (iii) adjustment for age, sex, education, diabetes duration, and random blood glucose (covariates which may impact on performance on screening tests for cognitive impairment). Since some covariates were missing for up to 6% of participants, involving 11 (9.9%) participants in total, missing values were multiply imputed (×5) before logistic regression modelling of the pooled dataset. Due to multiple bivariable comparisons, a two-tailed significance level of *p* < 0.010 was used throughout.

### 2.5. Sample Size

The hypothesis underlying the design of this study was that DFU in people with type 2 diabetes is associated with cognitive impairment. We therefore aimed to determine if there was an excess of cognitive impairment amongst people with type 2 diabetes with DFU compared to people with type 2 diabetes and no DFU. We planned to recruit 200 participants to the study—100 patients with DFU matched 1:1 to patients without DFU. An a priori power calculation was based on existing data from FDS2 which indicated that 8% of FDS2 participants with type 2 diabetes aged ≥50 years without a DFU had a baseline MMSE score <24 compared with 18% with a DFU. Assuming a correlation coefficient for cognitive impairment between matched groups of 0.04, we would be able to detect true two-sided odds ratios for cognitive impairment of 0.30 or 1.92 in patients with DFU relative to patients without DFU with 80% power and Type I error probability of 0.05. Due to logistic constraints, we recruited only 111 participants to the study and were able to detect true two-sided odds ratios for disease of 0.14 or 2.27.

## 3. Results

### 3.1. Characteristics of Clinic-Based Participants

The characteristics of participants recruited from MDFU and CDC are summarized in Table 1. There were 56 participants with type 2 diabetes and no DFU attending the CDC and 55 participants with type 2 diabetes with a DFU attending the MDFU clinic recruited to the study. MOCA and MMSE results were available for all study participants. The two groups had a similar age and sex distribution with a male predominance. There was no significant difference in diabetes duration. The MDFU group had a lower HbA_1c_ than the CDC group. The prevalence of macrovascular complications was similar in the two groups. The MDFU group also had an increased prevalence of self-reported and clinically-defined peripheral neuropathy. The prevalence of end-stage kidney disease was not significantly different between the two groups.

There were no significant differences between the two groups for MMSE and MOCA scores. There was also no significant difference between the proportions of participants having impaired cognition on either MOCA or MMSE after controlling for age, sex, education, diabetes duration, and random blood glucose (Table 2). There was a lower self-reported prevalence of depression and less use of antidepressant medication in the MDFU group. The MDFU group scored higher on NAFF but health literacy was similar.

### 3.2. MDFU and FDS2 Comparisons

The MDFU cohort had greater central adiposity assessed from waist circumference than the matched FDS2 sample (Table 3). There was no difference in HbA_1c_ between the two groups. The MDFU group had a greater prevalence of clinically-defined neuropathy as well as higher prevalence of foot ulcers and previous lower extremity amputation. There was no significant difference in MMSE score between the MDFU and FDS2 participants.

## 4. Discussion

The present study did not show an excess of cognitive impairment in patients with type 2 diabetes and DFU compared to patients with type 2 diabetes and no DFU when using assessment tools conventionally used in clinical practice. The median MMSE score was 28 and median MOCA score was 25 in the MDFU and CDC groups. Importantly, the groups were also similar in terms of potentially confounding comorbid conditions, including hypertension and prior cardiovascular disease, but the MDFU participants had significantly better glycemic control than those from CDCs, reported less depression, and had greater adherence to foot self-care. Although we did not find significant differences in these groups, there was a high proportion of patients with a MOCA score suggestive of at least mild cognitive impairment (≤25) in both the CDC (51.8%) and MDFU cohorts (56.4%). Median MMSE scores in the MDFU participants were also similar to those in a matched group of community dwelling people with type 2 diabetes from the FDS2 cohort. These findings question the proposition that cognitive impairment is over-represented in people with type 2 diabetes and DFU.

Only two other studies have compared cognition between patients with and without diabetes-related foot problems. The largest recruited almost 200 subjects and applied a computerized battery of neuropsychologic tests designed for the early detection of mild cognitive impairment and dementia [6]. Using this detailed evaluation, significantly greater baseline deficits in multiple cognitive domains, including memory, attention and concentration, reaction time, executive function, and psychomotor function, were identified in those with DFU. There were also significant differences between the two groups in education level, chronic diabetes complications, and HbA_1c_. However the differences in cognition remained after adjusting for these potential confounders. There was a significant decline in cognition from the premorbid state in the DFU group but not in participants without DFU. This study used a more sensitive instrument than the MMSE or MOCA but one that is not used in a usual care setting. Whether subtle cognitive impairment has implications for self-care behavior or adherence to foot care recommendations was not addressed in this study [6].

The second study, designed primarily to assess arterial stiffness and endothelial dysfunction in individuals with DFU, reported lower MMSE scores in patients with DFU compared to those with diabetes without DFU and when compared with healthy controls [17]. However, there was a significant imbalance of confounding risk factors with higher blood pressure, BMI, previous cardiovascular events, and dyslipidemia amongst patients with DFU. This complicates interpretation of the data.

There is only one other study with possibly relevant data. A small study of 30 patients requiring hospitalization for acute DFU management, most of whom had type 2 diabetes, reported a low average MOCA score of 22 [31]. However, there was no control group and hospitalization is itself associated with cognitive decrements [32]. There were no serial data that would have allowed an assessment of whether the cognitive deficit detected in the patients with DFU was transient or persisted after recovery from the acute illness.

The proportion of our participants recruited from hospital clinics with at least mild cognitive impairment based on MOCA scores (approximately half) is substantially higher than expected in the general population with reported prevalence rates of 11% in people over the age 60 years [22]. The CDC and MDFU groups in the present study were notable for their young age and may support the proposal of routine screening for cognitive impairment in this complex and vulnerable subgroup of patients with diabetes [22]. Furthermore, there is a likely need for educational programs designed for patients with DFU to be tailored for people with cognitive problems and may benefit from involving a caregiver where possible.

There are likely multiple underlying causes of cognitive impairment in patients with type 2 diabetes including diabetic retinopathy [19], hypertension [18], obesity, poor metabolic control, and, in particular, cerebrovascular disease [16]. On the other hand, peripheral neuropathy and lower extremity amputation do not appear to be associated with cognitive impairment [33,34]. The present data support the notion that any perceived cognitive deficit among patients with DFU may be a consequence of these comorbidities rather than being related to foot ulceration itself.

Multiple factors other than cognition contribute to patient adherence to recommended treatment regimens. The World Health Organisation multidimensional model of adherence classifies factors influencing adherence into five domains: patient-related, social- and economic-related, health system/health care team-related, therapy-related, and condition-related [35]. Other patient-related factors, such as health literacy or mood disorder, may also contribute to perceptions or understanding of health-care recommendations and willingness to engage in self-care. Poor health literacy is common amongst patients with type 2 diabetes [36] but two studies have shown no association between low health literacy and DFU or amputations [28,37]. Similarly, we found no differences in self-reported health literacy between participants with and without DFU. Some studies have suggested a high prevalence of depression among patients with DFU [38,39] but we found no difference in depression score on the PHQ4 between MDFU and CDC participants or FDS2 participants, and a higher prevalence of self-reported depression in the CDC participants than the MDFU participants. Patients’ illness beliefs have also been reported to be important determinants of foot self-care practices, however this was beyond the scope of this study [40].

An unexpected observation in the present study was that diabetes self-management and preventive care may have been better amongst the MDFU cohort compared to the CDC cohort. In comparison to previous studies of cognitive impairment in which patients with DFU had worse glycemic control and dyslipidemia, and higher blood pressure, glycated hemoglobin was lower in the MDFU cohort compared to the CDC cohort and there was no difference in blood pressure, or serum HDL- and LDL-cholesterol concentrations [6,17]. In addition, our patients with DFU had higher scores on the NAFF suggesting better protective foot self-care behavior amongst MDFU participants. This may simply be that the presence of an active DFU necessitates more attention to foot care behaviour but may also be due to intensive and regular MDFU clinic attendance and management.

The impact of cognitive impairment on wound-related outcomes, such as DFU healing time, LEA or DFU recurrence, is uncertain due to limited evidence. One study examining differences in cognition between individuals who had previously undergone LEA and those with foot ulcers without previous LEA found no differences between groups [34]. The study population was small, however, with only 20 participants in each group. A larger study including 56 participants with foot ulcers prospectively assessed incidence of foot ulcer relapse and found no relationship between relapse and cognition after adjusting for age, diabetes duration, and depression [41]. Future research should prospectively address the effect of cognition on wound healing, LEA, and DFU recurrence in people with diabetes.

The strengths of this study include the detailed clinical assessment evaluating not only cognition but also mood, self-care behavior, and health literacy. There was no imbalance between the MDFU and CDC cohorts in regards to blood pressure, lipid levels, and burden of comorbidities which may have complicated interpretation of cognitive data. The inclusion of an additional comparison with age-, sex-, and diabetes duration-matched FDS2 participants strengthens the finding that cognition was not significantly different between patients with DFU and those with diabetes and no foot ulcer, regardless of whether they are attending hospital outpatient clinics for management of complex type 2 diabetes or they are managed in the community setting.

The present study had limitations. Its cross-sectional design means that we are unable to draw conclusions regarding causality. Due to logistic constraints, we recruited 111 participants rather than the 200 participants originally planned and it is possible this may have contributed to a type II error. However, the difference between the groups was in the opposite direction to that we had hypothesised, with less people with type 2 diabetes with a DFU having a MMSE score ≤ 24 than people with type 2 diabetes and no DFU. This suggests strongly that a larger sample size would not have provided data supporting our original research hypothesis. The relatively small sample size also constrained the number of variables included in multivariable logistic regression, but our cohorts were similar at baseline for important potential confounders, including diabetes duration, smoking status, BMI, blood pressure, lipid profile, previous myocardial infarction, and stroke. Recruitment from hospital outpatient clinics may limit the generalizability of our findings to DFU patients being managed in the community setting, and raises the possibility of selection of a more motivated and engaged subpopulation of patients. Findings such as better glycaemic control, less self-reported depression, and greater adherence to foot self-care amongst the MDFU cohort compared to the CDC cohort may not be generalizable to patients with DFU not managed in an intensive multidisciplinary setting. Comparisons between the MDFU clinic-based cohort and FDS2 community-based cohort may potentially be biased due to the different participant recruitment settings. Assessment of foot self-care relied on self-reporting, allowing for the possibility of recall and social desirability bias. Finally, no longitudinal data were assessed and so the prognostic impact of any differences between cohorts could not be investigated. Future prospective longitudinal studies may help to clarify the clinical impact of cognition on DFU healing.

In conclusion, cognitive impairment assessed using readily available clinical tools does not appear to be more common in patients with diabetes with DFU than in those who do not. Other patient-related factors potentially affecting adherence such as health literacy and depression score also did not differ between cohorts. Non-adherence and adverse DFU outcomes may potentially be driven by other factors such as socio-economic disadvantage, health system inequity, and complexity of treatment regimens rather than impaired cognition [10]. However, approximately half of participants recruited from hospital CDC or MDFU clinics had at least mild cognitive impairment (based on MOCA scores) which may support screening for cognitive impairment in patients with DFU and/or tailoring of foot education interventions to account for potential cognitive problems.

## Figures and Tables

**Table 1:** Characteristics of participants attending the Complex Diabetes Clinic (CDC) versus the Multidisciplinary Diabetes Foot Ulcer Clinic (MDFUC).

	CDC Attendees	MDFUC Attendees	*p*-Value
*N* (%)	56 (50.5)	55 (49.5)	
Age (years)	61.6 ± 10.6	63.6 ± 12.9	0.39
Sex (% male)	57.1	74.5	0.07
Overseas born (%)	58.9	34.5	0.013
Birth place (%):			0.001
Australia/NZ	46.4	69.1	
UK/Europe	25.0	29.1	
Asia	16.1	1.8	
Other	12.5	0	
Education (% secondary/tertiary)	53.6/46.4	60.0/40.0	0.57
Marital status (%)			0.018
Single	23.2	34.5	
Married/de facto	48.2	54.5	
Divorced/separated	23.2	3.6	
Widowed	5.4	7.3	
Smoking status (%):			0.49
Never	41.1	36.4	
Ex-	46.4	56.4	
Current	12.5	7.3	
Alcohol (standard drinks/week)	0 [0–1]	0 [0–2]	0.70
Vigorous exercise (%)	35.7	36.4	>0.99
BMI (kg/m^2^)	34.5 ± 7.0	34.3 ± 8.5	0.86
Central adiposity (% by waist circumference)	92.7	100	0.12
ABSI * 10^3^ (m^11/6^ kg^−2/3^)	82.8 ± 5.6	86.2 ± 5.3	0.001
Heart rate (bpm)	76 ± 11	78 ± 13	0.32
Systolic blood pressure (mm Hg)	138 ± 16	144 ± 19	0.12
Diastolic blood pressure (mm Hg)	77 ± 11	76 ± 16	0.77
On antihypertensive medication (%)	78.6	87.3	0.31
Self-reported hypertension (%)	49.4	50.6	0.83
Age at DM diagnosis (years)	46.8 ± 10.3	46.0 ± 14.7	0.77
Duration DM (years)	15.0 [7.0–20.0]	17.5 [10.0–24.0]	0.26
Random glucose (mmol/L)	9.4 [7.5–13.3]	10.1 [8.1–13.5]	0.43
HbA_1c_ (%)	8.6 [7.6–9.7]	7.6 [6.5–8.9]	0.001
HbA_1c_ (mmol/mol)	70.5 [59.6–82.5]	59.6 [47.5–73.8]	0.001
Diabetes treatment (%):			0.021
Diet	0	1.8	
Oral glucose lowering medications (OGLMs) ± non-insulin injectables	14.3	32.7	
Insulin only	10.7	16.4	
Insulin ± OGLMs ± non-insulin injectables	75.0	49.1	
Total cholesterol (mmol/L)	4.2 ± 1.1	4.0 ± 1.4	0.39
HDL-cholesterol (mmol/L)	1.06 ± 0.36	1.00 ± 0.32	0.43
LDL-cholesterol (mmol/L)	1.9 ± 0.9	2.1 ± 1.0	0.59
Serum triglycerides (mmol/L)	2.3 (1.2–4.6)	1.8 (1.0–3.3)	0.037
Lipid-modifying treatment (%)	78.6	80.0	>0.99
Self-reported dyslipidaemia (%)	80.4	70.9	0.28
Aspirin (%)	44.6	49.1	0.71
Other antiplatelets (%)	8.9	25.5	0.025
Self-reported retinopathy (%)	14.3	32.7	0.026
Urinary albumin:creatinine ratio (mg/mmol)	5.3 (0.8–32.7)	11.3 (1.8–70.8)	0.042
Albuminuria (%):			0.14
Normal (<3.0 mg/mmol)	51.9	34.0	
Micro- (3.0–29.9 mg/mmol)	32.7	38.0	
Macro- (≥30.0 mg/mmol)	15.4	28.0	
On ACE-I/ARB (%)	64.3	70.9	0.54
eGFR (CKD-EPI) categories (%):			0.09
≥90 mL/min/1.73 m^2^	38.2	23.6	
60–89 mL/min/1.73 m^2^	34.5	32.7	
45–59 mL/min/1.73 m^2^	5.5	21.8	
30–44 mL/min/1.73 m^2^	10.9	7.3	
<30 mL/min/1.73 m^2^	10.9	14.5	
eGFR (CKD-EPI) < 60 mL/min/1.73 m^2^ (%):	27.3	43.6	0.11
Self-reported kidney replacement treatment (%)	1.8	10.9	0.06
ESKD (%)	3.6	12.7	0.16
Self-reported neuropathy (%)	60.7	92.7	<0.001
Clinical neuropathy (%)	57.1	98.2	<0.001
Abnormal Foot Appearance (%; either side)	92.9	98.2	0.36
Prevalent or recently healed DFU (%)	1.8	100	<0.001
Gangrene (%)	1.8	20.0	0.002
Previous LEA (%)	5.4	63.0	<0.001
ABI (either side) < 0.9 (%)	10.9	14.8	0.58
Self-reported PVD (%)	7.1	21.8	0.033
Self-reported MI (%)	25.0	25.5	>0.99
Self-reported AF (%)	1.8	14.5	0.016
Self-reported stroke (%)	3.6	10.9	0.16
Self-reported cancer (%)	10.7	12.7	0.78
Nottingham Assessment of Functional Footcare (NAFF)	50 [43–55]	57 [53–63]	<0.001
MMSE score	28 [27–29]	28 [27–29]	0.90
MMSE ≤ 24 (%)	10.7	3.6	0.27
MOCA score	25 [24–27]	25 [22–27]	0.72
MOCA ≤ 25 (%)	51.8	56.4	0.71
Self-reported depression (%)	60.7	30.9	0.002
Self-reported anxiety (%)	44.6	21.8	0.015
PHQ4 total	4 [1–6]	2 [0–4]	0.022
PHQ4 (% normal/mild/moderate/severe)	37.5/30.4/19.6/12.5	63.6/18.2/9.1/9.1	0.048
PHQ4 depression score	2 [0–4]	1 [0–2]	0.08
PHQ4 depression score ≥ 3 (%)	30.4	23.6	0.52
PHQ4 anxiety score	2 [0–4]	1 [0–2]	0.026
PHQ4 anxiety score ≥ 3 (%)	33.9	16.4	0.048
On antidepressant medication (%)	42.9	16.4	0.003
Health literacy	13 [10–15]	13 [12–15]	0.32

Data are shown as percentages (%), mean ± SD, median [IQR], or geometric mean (SD range).

**Table 2:** Logistic regression models with cognition as the outcome and Multidisciplinary Diabetes Foot Ulcer Clinic (MDFUC) vs Complex Diabetes Clinic (CDC) attendance the exposure of interest.

Dependent Variable	Independent Variables	OR (95% CI)	*p*-Value
MMSE < 25	Clinic (reference CDC)	0.31 (0.06–1.63)	0.17
	Age (increase of one year)	1.05 (0.98–1.13)	0.14
	Sex (reference female)	0.94 (0.20–4.37)	0.93
	Clinic (reference CDC)	0.26 (0.05–1.46)	0.13
	Age (increase of one year)	1.09 (1.002–1.18)	0.044
	Sex (reference female)	0.47 (0.08–2.81)	0.41
	Education (reference tertiary)	1.28 (0.20–8.05)	0.79
	Diabetes duration (increase of one year)	0.91 (0.81–1.02)	0.11
	Random blood glucose (increase of 1 mmol/L)	1.11 (0.98–1.26)	0.10
	Clinic (reference CDC)	0.24 (0.04–1.58)	0.14
MOCA < 26	Clinic (reference CDC)	1.20 (0.57–2.54)	0.63
	Age (increase of one year)	1.03 (0.99–1.06)	0.12
	Sex (reference female)	0.85 (0.38–1.92)	0.70
	Clinic (reference CDC)	1.18 (0.55–2.56)	0.67
	Age (increase of one year)	1.03 (0.99–1.07)	0.15
	Sex (reference female)	0.86 (0.36–2.05)	0.74
	Education (reference tertiary)	0.84 (0.38–1.87)	0.67
	Diabetes duration (increase of one year)	1.01 (0.96–1.05)	0.74
	Random blood glucose (increase of 1 mmol/L)	1.03 (0.96–1.11)	0.42
	Clinic (reference CDC)	1.16 (0.53–2.56)	0.71

**Table 3:** Characteristics of age-, sex-, and diabetes duration-matched FDS2 participants with type 2 diabetes versus participants attending the MDFUC.

	Matched FDS2 Participants	MDFUC Attendees	*p*-Value
*N* (%)	55 (50.0)	55 (50.0)	
Age (years)	63.3 ± 12.8	63.6 ± 12.9	0.91
Sex (% male)	74.5	74.5	>0.99
Duration DM (years)	15.8 [9.8–23.0]	17.5 [10.0–24.0]	0.80
Overseas born (%)	41.8	34.5	0.56
Birth place (%):			0.17
Australia/NZ	60.0	69.1	
UK/Europe	27.3	29.1	
Asia	5.5	1.8	
Other	7.3	0	
Not fluent in English (%)	7.3	0	0.12
Education (% primary/secondary/tertiary)	14.8/48.1/37.0	0/60.0/40.0	0.009
Marital status (%)			0.12
Single	16.4	34.5	
Married/de facto	61.8	54.5	
Divorced/separated	9.1	3.6	
Widowed	12.7	7.3	
Smoking status (%):			0.24
Never	40.0	36.4	
Ex-	43.6	56.4	
Current	16.4	7.3	
Alcohol (standard drinks/week)	0.1 [0–1]	0 [0–2]	0.17
BMI (kg/m^2^)	31.2 ± 6.2	34.3 ± 8.5	0.031
Central adiposity (% by waist circumference)	70.4	100	<0.001
ABSI * 10^3^ (m^11/6^ kg^−2/3^)	82.7 ± 5.3	86.2 ± 5.3	0.001
Heart rate (bpm)	73 ± 16	78 ± 13	0.09
Systolic blood pressure (mm Hg)	148 ± 21	144 ± 19	0.28
Diastolic blood pressure (mm Hg)	80 ± 14	76 ± 16	0.18
On antihypertensive medication (%)	76.4	87.3	0.22
Age at DM diagnosis (years)	46.5 ± 14.6	46.0 ± 14.7	0.86
HbA_1c_ (%)	7.1 [6.3–8.6]	7.6 [6.5–8.9]	0.30
HbA_1c_ (mmol/mol)	54.1 [45.4–70.5]	59.6 [47.5–73.8]	0.30
Diabetes treatment (%):			0.029
Diet	7.3	1.8	
Oral glucose lowering medications (OGLMs) ± non-insulin injectables	52.7	32.7	
Insulin only	5.5	16.4	
Insulin ± OGLMs ± non-insulin injectables	34.5	49.1	
Total cholesterol (mmol/L)	4.4 ± 1.1	4.0 ± 1.4	0.14
HDL-cholesterol (mmol/L)	1.16 ± 0.24	1.00 ± 0.32	0.005
LDL-cholesterol (mmol/L)	2.3 ± 0.8	2.1 ± 1.0	0.15
Serum triglycerides (mmol/L)	1.7 (0.9–3.0)	1.8 (1.0–3.3)	0.55
Lipid-modifying treatment (%)	70.9	80.0	0.38
Aspirin (%)	34.5	49.1	0.18
Urinary albumin:creatinine ratio (mg/mmol)	4.4 (0.8–25.4)	11.3 (1.8–70.8)	0.010
Albuminuria (%):			0.11
Normal (<3.0 mg/mmol)	50.9	34.0	
Micro- (3.0–29.9 mg/mmol)	35.8	38.0	
Macro- (≥30.0 mg/mmol)	13.2	28.0	
On ACE-I/ARB (%)	67.3	70.9	0.84
eGFR (CKD-EPI) categories (%):			0.10
≥90 mL/min/1.73 m^2^	40.7	23.6	
60–89 mL/min/1.73 m^2^	38.9	32.7	
45–59 mL/min/1.73 m^2^	13.0	21.8	
30–44 mL/min/1.73 m^2^	1.9	7.3	
<30 mL/min/1.73 m^2^	5.6	14.5	
eGFR (CKD-EPI) < 60 mL/min/1.73 m^2^ (%):	20.4	43.6	0.013
Kidney replacement treatment (%)	1.8	10.9	0.11
ESKD (%)	5.5	12.7	0.32
Clinically-defined neuropathy * (%)	59.3 (MNSI > 2)	98.2	<0.001
Prevalent or recently healed DFU (%)	9.3	100	<0.001
Previous LEA (%)	1.8	63.0	<0.001
ABI (either side) < 0.9 (%)	16.7	14.8	>0.99
MMSE score for age ≥ 50 years	28 [26–30] (n = 36/45)	28 [26–29] (n = 46/46)	0.73
MMSE ≤ 24 (%) for age ≥ 50 years	16.7	4.3	0.13
PHQ4 depression score	0 [0–2] (n = 47)	1 [0–2]	0.06
PHQ4 depression score ≥ 3 (%)	12.8 (n = 47)	23.6	0.21
On antidepressant medication (%)	10.9	16.4	0.58

Data Percentages (%), mean ± SD, median [IQR], or geometric mean (SD range). * Clinically-defined neuropathy was defined in FDS2 using the Michigan Neuropathy Screening Instrument (MNSI > 2) and in the DFU cohort via clinical examination (peripheral sensory neuropathy was determined to be present if loss of protective sensation was detected using the 10 g monofilament and/or reduced sensation to light touch was detected in one or both lower limbs).

## Data Availability

All relevant data is contained within the article.
